# Practical recommendations for the allergological risk assessment of the COVID-19 vaccination – a harmonized statement of allergy centers in Germany 

**DOI:** 10.5414/ALX02225E

**Published:** 2021-01-26

**Authors:** Margitta Worm, Andrea Bauer, Bettina Wedi, Regina Treudler, Wolfgang Pfuetzner, Knut Brockow, Timo Buhl, Torsten Zuberbier, Joachim Fluhr, Gerda Wurpts, Ludger Klimek, Thilo Jakob, Hans F. Merk, Norbert Mülleneisen, Stefani Roeseler, Heinrich Dickel, Ulrike Raap, Jörg Kleine-Tebbe

**Affiliations:** 1 Allergology and Immunology, Department of Dermatology, Venereology and Allergology, Campus Charité Mitte, University Medicine Berlin,; 2 Clinic and Polyclinic for Dermatology, University Hospital Carl Gustav Carus at the Technical University Dresden,; 3 Department of Dermatology, Allergology and Venereology Comprehensive Allergy Center (CAC) Treatment Center for Hereditary Angioedema in the ZSE, Hannover Medical School,; 4 Clinic of Dermatology, Venereology and Allergology, Leipzig University Medical Center,; 5 Hesse Allergy Center, Clinic for Dermatology and Allergology, Marburg University Hospital,; 6 Dermatology Clinic Campus Biederstein, Klinikum rechts der Isar, Technical University of Munich,; 7 Dermatology Venereology and Allergology Clinic, University Medical Center Göttingen Georg-August-University,; 8 Department of Dermatology and Allergy, Comprehensive Allergy Center Charité – Universitätsmedizin Berlin, Germany, corporate member of Freie Universität Berlin, Humboldt-Universität zu Berlin and Berlin Institute of Health,; 9 Department of Dermatology and Allergology, University Hospital Aachen,; 10 Center for Rhinology and Allergology of the ENT University Clinic Mannheim, Wiesbaden,; 11 Department of Dermatology, Venerology and Allergology, University Hospital Giessen,; 12 Dermatology Clinic, RWTH Aachen University,; 13 Asthma and Allergy Center, Leverkusen,; 14 Allergy, Asthma and Anaphylaxis Center, Clinic of Pneumology, Allergology, Sleep and Respiratory Medicine, Augustinians Hospital, Cologne,; 15 Department of Allergology, Occupational and Environmental Dermatology, Clinic for Dermatology, Venereology and Allergology, St. Josef Hospital, Ruhr University Bochum,; 16 Department of Experimental Allergology and Immunodermatology, Department of Human Medicine, University of Oldenburg, and; 17 Allergy and Asthma Center Westend, Berlin, Germany

**Keywords:** COVID-19 vaccination, allergy, anaphylaxis, polyethylene glycol, polysorbate 80, skin test

## Abstract

Severe allergic reactions to vaccines are very rare. Single severe reactions have occurred worldwide after vaccination with the new mRNA-based COVID-19 vaccines. PEG2000 is discussed as a possible trigger. We provide guidance on risk assessment regarding COVID-19 vaccination in patients with allergic diseases and suggest a standardized, resource-oriented diagnostic and therapeutic procedure. Reports of severe allergic reactions in the context of COVID-19 vaccination can be made via www.anaphylaxie.net using an online questionnaire.

## Introduction 

Vaccination is a very efficient method to prevent pathogen-related diseases. Smallpox vaccination, for example, has helped to completely eradicate this potentially life-threatening disease. In Germany, the current vaccination recommendations for the population are issued by the Standing Commission on Vaccination (StiKo) and are updated regularly [1]. Severe allergic vaccination reactions are very rare and can be caused by the vaccine itself (very rare) or the ingredients (adjuvants, antibiotics, hen’s egg, carriers, preservatives) of the vaccine solution [2]. The incidence of severe immediate allergic reactions after vaccination varies from 1 : 100,000 to 1 : 1 million depending on the vaccine and the population studied [2]. Since the outbreak of the COVID-19 pandemic, several vaccines have been developed worldwide, with two mRNA-based products licensed in Europe to date. An additional adenovirus-based vaccine is currently in use in the United Kingdom (Table 1). 

Since the approval of the mRNA-based vaccines, several case series have been published from the United Kingdom and the United States regarding individuals who have experienced severe general reactions in the setting of COVID-19 vaccination [3]. From December 14 – 23, 2020, surveillance by the Vaccine Adverse Event Reporting System identified 21 cases of anaphylaxis after administration of 1,893,360 first doses of Pfizer-BioNTech COVID-19 vaccine (11.1 cases per million doses). In Germany, the Paul Ehrlich Institute (PEI) has received reports of 17 anaphylactic reactions as of January 10, 2021 [4]. All individuals reported to date with adverse events related to COVID-19 vaccination due to suspected allergy or intolerance have survived the reaction without harm. 

Polyethylene glycol (PEG) is discussed as the trigger of the reactions [5]. It is bound to a liposomal matrix, which is a nanoparticle that coats the viral mRNA of the COVID-19 vaccine. The PEG with a molecular weight of 2,000 (PEG2000) serves as a stabilizer to prevent premature degradation of the nanoparticles by the mononuclear phagocytosis system, also as a solubilizer during the transition of the particles into the intracellular cytosol due to its hygroscopic properties, and as an adjuvant due to its immunogenic potential [6]. PEGs are produced via polymerization of ethylene oxide. Another name for PEG is macrogol. Possible cross-reactions to polysorbate 80 (polyoxyethylene-20-sorbitan monooleate, low molecular weight PEG, 1,310 Dalton, CAS 9005-65-6, Tween 80 and E-433), which has recently been identified primarily as an anaphylaxis inducer after administration of biologicals, must be considered [7, 8, 9]. 

Hypersensitivity to PEG is very rare, especially considering its widespread use in numerous everyday products (cosmetics, medications, laxatives, lozenges) [10, 11, 12, 13]. Various allergic manifestations such as late reactions like allergic contact dermatitis, but also contact urticaria and anaphylaxis as immediate reactions have been described. In the anaphylaxis registry, 6 PEG cases and 1 polysorbate case have been registered so far, resulting in a percentage for PEG and polysorbate of 0.3% (7/2,350) for drug-induced anaphylaxis [14]. 

## Practical recommendations 

After the British authority issued a warning regarding the use of the vaccine in patients with severe allergies following the occurrence of 2 severe allergic reactions after vaccination, there were critical statements from the allergy societies and subsequently more concrete assessments from the European Medicines Agency (EMA) and the PEI, respectively, regarding a possible risk potential [15]. They conclude that so far there is no evidence that patients with allergies in general have an increased risk of vaccination. Therefore, building on the currently available information, a graded risk assessment was recently proposed by the guideline group “Management of Anaphylaxis” (Table 2). 

This states that for a defined group of patients, further allergological clarification is useful before COVID-19 vaccination. In the following, we would like to present the most important diagnostic steps for the allergological assessment of the tolerability of the COVID-19 vaccine and the resulting therapeutic consequences. 

## Patient history 

The following questions are relevant for the assessment of the allergological risk potential for COVID-19 vaccination:

- Have there been previous severe allergic reactions to medications, vaccines, or PEG-macrogol or polysorbate-containing drugs (e.g., macrogol or cold medications).

If yes, did these reactions occur repeatedly and to which preparations?

- Have there been (repeated) severe general reactions during medical procedures such as colonoscopies, operations under general anesthesia?
- Has a severe general reaction to an unknown trigger ever occurred?
- Is a mastocytosis known in patients with previous severe immediate drug reactions or anaphylaxis to an unknown trigger?
- In case of second COVID-19 vaccine administration: has a severe general reaction occurred after administration of the first dose?

## Diagnostic clarification and therapeutic consequence 

**Figure 1.**
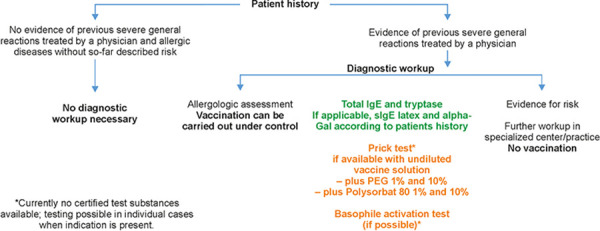
Proposed diagnostic allergological procedure in case of a possible vaccination risk assessment.

The allergological diagnostic work-up (Figure 1) includes, after a thorough history, the determination of basal tryptase, total IgE, and sIgE (depending on the history e.g. of latex, ethylene oxide, α-Gal or gelatine, CCD). Certified test methods for the determination of IgE antibodies against PEG2000 (or IgM antibodies), which are thought to play a role in triggering complement-mediated hypersensitivity reactions to PEG [16], are currently not available. A basophil activation test can be considered, but again, no certified and validated test systems are currently available. 

Individual testing of a given patient may be considered for skin testing, which should be performed in a facility familiar with the management of severe allergic reactions. Since there are no approved test substances, a prick test can be performed with the respective vaccine solution (pure, if available – e.g., residual vaccine) as well as with PEG2000 and polysorbate 80 (titrated 10% and 1%). However, it is not yet known whether a negative prick test actually has sufficient positive or negative predictive power for a given vaccine to determine the risk of a severe systemic general reaction. Intracutaneous testing beyond this, and only if the prick tests are negative, can be considered after critically weighing the benefits and risks, with substances that can be used for this purpose. In the literature, severe, and in individual cases fatal, allergic reactions have been reported during the course of intracutaneous testing [10]. 

The systemic immediate reactions reported so far in the context of COVID-19 vaccination may be caused by different pathomechanisms. Besides IgE-mediated reactions, non-IgE-mediated mechanisms via the MAS-related G protein-coupled receptor-X2 (MRGPRX2) or complement-dependent activation pathways (complement activation-related pseudoallergy = CARPA) may play a role which cannot necessarily be detected by means of a prick test. The indication for intracutaneous testing should therefore be made strictly as described above, since firstly severe allergic reactions can occur during testing and secondly the potential risk of inducing a sensitization. Currently, a possible extended skin testing protocol is being developed in the certified allergy centers of the DGAKI (CAC) in cooperation with allergology focus centers. 

If all tests are negative, a short-term vaccination may be necessary (e.g., personnel in COVID -19 intensive care units) from an allergological point of view, taking appropriate precautions (emergency medication and trained personnel available, monitoring for at least 30 minutes after vaccination). If a positive result, e.g., for PEG, is found in the skin test, another vaccine can be considered for vaccination, provided that the vaccine is available (within a reasonable time). It should be noted that polysorbate 80, which is commonly found in influenza vaccines, for example, and is also present in Astra Zeneca’s non-mRNA-based vaccine (less than 100 μg/dose), is potentially cross-reactive to PEGs [7] and may also cause anaphylactic reactions [8, 9]. In the United Kingdom, it has been recommended that patients with PEG allergy can receive Astra-Zeneca’s vaccine under 30 minutes of observation [17], whereas in the United States, the CDC most recently classified a previous reaction to polysorbate 80 as a contraindication to an mRNA-based vaccine [18]. 

## Funding 

None. 

## Conflict of interest 

None. 

**Table 1.** Ingredients of the vaccines discussed in the text.

**Astra Zeneca AZD1222**	**BioNTech BNT162b2**	**Moderna mRNA-1273**
- L-Histidine - L-Histidine hydrochloride monohydrate - Magnesium chloride hexahydrate - Polysorbate 80 - Ethanol - Sucrose - Sodium chloride - Disodium edetate dihydrate - Water for injections	- ((4-hydroxybutyl)azanediyl) bis (hexane-6,1-diyl)bis(2-hexyldecanoate) (ALC-0315) - 2-[(polyethylene glycol)-2000]-N,N-ditetradecylacetamide (ALC-0159) - 1,2-Distearoyl-sn-glycero-3-phosphocholine (DSPC) - Cholesterol - Potassium chloride - Potassium dihydrogen phosphate - Sodium chloride - Disodium phosphate dihydrate - Sucrose - Water for injections	- SM-102, 1,2-dimyristoylrac-glycero-3-methoxypolyethylene glycol-2000 [PEG2000-DMG] - Cholesterol - 1,2-distearoyl-sn-glycero-3-phosphocholine (DSPC) - Tromethamine (-HCI) - Acetic acid - Sodium acetate - Sucrose

**Table 2.** Allergic risk assessment for COVID-19 vaccination. Modified from Worm et al., MMW 2021 [19].

**Vaccination unproblematic from the current allergological point of view**	Vaccination with increased risk awareness* or preceding allergic workup	**No vaccination according to summary of product characteristics and from an allergological point of view**
- Allergic asthma - Allergic rhinoconjunctivits - Atopc eczema - Food allergy - Insect venom allergy - Allergic contact eczema - Urticaria - History of drug eruption - Delayed local reactions to vaccinations	Anaphylaxis treated by a physician - during a vaccination or medical procedure like coloscopy, surgical intervention under general anesthesia - after (repeated) drug intake - of unclear pathogenesis	History of severe allergic reactions to one or several of the vaccine ingredients

*Whether mastocytosis per se is associated with an increased risk of vaccination has not been clearly established to date.
